# Primary Neuronal Precursors in Adult Crayfish Brain: Replenishment from a Non-neuronal Source

**DOI:** 10.1186/1471-2202-12-53

**Published:** 2011-06-02

**Authors:** Jeanne L Benton, Yi Zhang, Colleen R Kirkhart, David C Sandeman, Barbara S Beltz

**Affiliations:** 1 Neuroscience Program, Wellesley College, 106 Central Street, Wellesley, MA 02481, USA

## Abstract

**Background:**

Adult neurogenesis, the production and integration of new neurons into circuits in the brains of adult animals, is a common feature of a variety of organisms, ranging from insects and crustaceans to birds and mammals. In the mammalian brain the 1^st^-generation neuronal precursors, the astrocytic stem cells, reside in neurogenic niches and are reported to undergo self-renewing divisions, thereby providing a source of new neurons throughout an animal's life. In contrast, our work shows that the 1^st^-generation neuronal precursors in the crayfish (*Procambarus clarkii*) brain, which also have glial properties and lie in a neurogenic niche resembling that of vertebrates, undergo geometrically symmetrical divisions and *both *daughters appear to migrate away from the niche. However, in spite of this continuous efflux of cells, the number of neuronal precursors in the crayfish niche continues to expand as the animals grow and age. Based on these observations we have hypothesized that (1) the neuronal stem cells in the crayfish brain are not self-renewing, and (2) a source external to the neurogenic niche must provide cells that replenish the stem cell pool.

**Results:**

In the present study, we tested the first hypothesis using sequential double nucleoside labeling to track the fate of 1^st^- and 2^nd^-generation neuronal precursors, as well as testing the size of the labeled stem cell pool following increasing incubation times in 5-bromo-2'-deoxyuridine (BrdU). Our results indicate that the 1^st^-generation precursor cells in the crayfish brain, which are functionally analogous to neural stem cells in vertebrates, are not a self-renewing population. In addition, these studies establish the cycle time of these cells. In vitro studies examining the second hypothesis show that Cell Tracker™ Green-labeled cells extracted from the hemolymph, but not other tissues, are attracted to and incorporated into the neurogenic niche, a phenomenon that appears to involve serotonergic mechanisms.

**Conclusions:**

These results challenge our current understanding of self-renewal capacity as a defining characteristic of all adult neuronal stem cells. In addition, we suggest that in crayfish, the hematopoietic system may be a source of cells that replenish the niche stem cell pool.

## Background

It is now well established that neurogenesis occurs not only during embryogenesis and early postembryonic development, but also in the brains of many adult vertebrates and non-vertebrates, including in the human hippocampus and olfactory bulb [1]. The production of adult-born neurons is regulated by a variety of factors such as environmental enrichment, diet, circadian signals, serotonin, and nitric oxide [2,3]. Studies suggest that the new neurons may play a role in learning and memory [4], and many diseases have also been linked to a dysregulation of adult neurogenesis [5].

Neurogenic niches are specialized vascularized microenvironments containing stem cells that serve as the 1^st ^generation neuronal progenitors in the mammalian brain [6]. In the adult subventricular (SVZ) and subgranular (SGZ) zones in rodents, relatively quiescent neural stem cells with glial properties [7] give rise to more rapidly proliferative transit amplifying cells; these expand the progenitor pool and produce neuroblasts that divide yet again. The SVZ generates neuroblasts that migrate towards the olfactory bulb where they differentiate into olfactory interneurons, while the SGZ generates cells that become hippocampal neurons [8]. The neural stem cells that initiate these lineages are proposed to be slowly dividing, self-renewing populations. However, due to the complexity of mammalian neurogenic niches where several precursor cell generations coexist, there is limited in vivo evidence for these properties [9,10].

**Figure 1:**
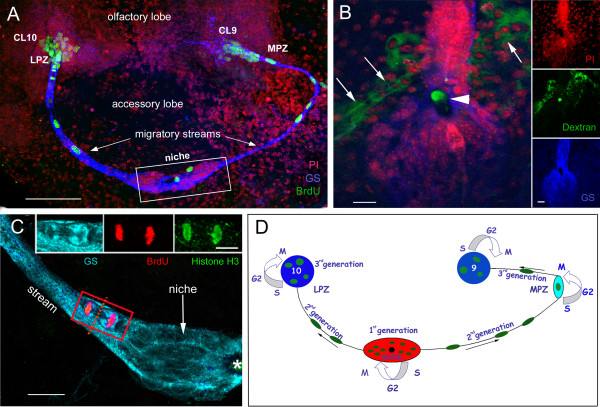
**Neurogenesis in the adult crayfish brain**. **A**. Crayfish were exposed to BrdU for 6 hours followed by immediate sacrifice. Confocal image of a crayfish hemi-brain labeled immunocytochemically for BrdU (green) and glutamine synthetase (blue) and counterstained with propidium iodide (red), a marker of nucleic acids. BrdU-labeled cells are observed within both the lateral (LPZ) and medial (MPZ) proliferation zones. Precursor cells located in a niche (rectangle) generate daughter cells that migrate along the streams to either the LPZ or MPZ. **B**. Dextran-fluorescein (green) injections into the pericardial sinus show that the "vascular cavity" of the niche (arrowhead) is confluent with the vasculature. The niche lies on a blood vessel (arrows). Propidium iodide (red), and glutamine synthetase (blue) labeling are also shown. Sidebar shows each channel separately. **C**. A triple-labeled M-phase cell close to the niche is immunolabeled with glutamine synthetase (cyan), phophohistone-H3 (green) and BrdU (red). The asterisk marks the vascular cavity. [Image from 17] **D**. Model summarizing events leading to the production of olfactory and accessory lobe interneurons in adult crayfish brain [Image from 29]. 1^st ^generation precursors reside within a neurogenic niche where they divide symmetrically. Both daughter cells appear to migrate to the proliferation zones towards either the LPZ or the MPZ. At least one more division will occur in the LPZ and MPZ before the progeny (third- and subsequent-generations) differentiate into neurons. Black arrows indicate direction of precursor cell migration; curved arrows indicate locations of cell divisions. Scale bars: A, 100 μm; B, 20 μm, side bar 20 μm; C, 20 μm; C inset, 10 μm.

In the crayfish *Procambarus clarkii*, 1^st^-generation neuronal precursors that have glial properties are located in a niche on the ventral surface of the brain, midway between two proliferation zones (lateral, LPZ; medial, MPZ) where neuronal differentiation occurs (Figure 1A) [11]. The niche cells label with an antibody generated against glutamine synthetase (GS), an enzyme that converts glutamate to glutamine, and which is also a marker of astrocytes and early stem cells in the vertebrate brain [12,13]. When labeled immunocytochemically for GS, the entire system generating neurons in the adult crayfish brain is revealed (Figure 1A) [11,14]. As in mammals, the crayfish neurogenic niche is intimately associated with the vasculature, as it sits on a blood vessel that is confluent with the niche via a vascular cavity (Figure 1B) (see also [11]). The 1^st^-generation cells divide relatively slowly and their daughters migrate along streams (Figure 1A, D) formed by processes of the niche cells [11]. Thus, these 1^st^-generation cells in the crustacean brain function as both precursor and support cells. The migratory 2^nd^-generation precursor cells require 5-7 days to traverse the streams [11]. After these reach the proliferation zones in cell cluster 9 or 10, they divide at least once more; their daughters differentiate into interneurons innervating olfactory and higher order processing regions, the olfactory and accessory lobes, respectively [11,15,16].

Many aspects of this sequence leading to the production of neurons in the adult crayfish brain are reminiscent of adult neurogenesis in the mammalian brain, suggesting that adult neurogenesis may be governed by common ancestral mechanisms that have been retained in a phylogenetically broad group of species, or, alternatively, reflect convergence on common mechanisms. However, in contrast to the intermingling of precursor cell generations in the mammalian neurogenic niche, the 1^st^-generation precursors comprising the niche in crayfish are segregated from their migratory daughters, which in turn are separated from their descendants residing in the proliferation zones. As a result of this compartmentalization of the lineage, the relationships among the precursors is clear and quantitative changes in each generation are easily assessed.

Studies exploring the mode of division of the 1^st^-generation precursors in crayfish revealed that these cells undergo geometrically symmetrical divisions (Figure 1C) [17]. These divisions often occur in the niche near where the streams emerge, or in the initial segments of the stream. The cytoplasm of the dividing/migratory cells is GS-labeled (Figure 1C, inset), a characteristic feature of the niche cells, thus confirming the ancestry of these cells, although it is not known if all niche cells are competent to become neuronal precursors. At any given time, the majority of niche cells are in G_1 _of the cell cycle [11]. The fact that these divisions often occur in the proximal parts of the migratory stream and that pairs of cells are frequently observed along the migratory route, indicates that both daughter cells exit from the niche. These data therefore suggest that the outcome of these mitoses is unlike the self-renewing divisions that are generally associated with stem cells where one daughter remains at the site of division to maintain the stem cell pool and the other pursues a different fate.

In the present study, we calculated the cycle time of the 1^st^-generation niche precursor cells, confirming that these are a slowly dividing population (cycle time >48 hrs). Further, we have directly tested the self-renewal capacity of the 1^st^-generation niche precursor cells. The results of these studies confirm our original suspicion that these neuronal precursors are not self-renewing, although as the cells that initiate the lineage that produces adult-born neurons, they serve a function that is analogous to the stem cells in the mammalian niche. These data raise crucial questions, because the niche population is known to expand as the animals grow and age over several years, while the length of the precursor cell cycle increases [17]. Why isn't the supply of 1^st^-generation precursor cells in the niche depleted over time, as they divide and their daughters migrate away? By what mechanisms do the number of niche cells expand as the animals grow? Our data strongly indicate that neuronal precursors are recruited to the niche from an extrinsic source. Moreover, in vitro experiments show that, among several cell types, those harvested from the hemolymph are uniquely attracted to the niche and that this affinity may involve serotonergic mechanisms. Taken together, these data suggest that the hematopoietic system may be a source of cells that can replenish the niche cell population as these 1^st^-generation neuronal precursors divide and their daughters migrate to the proliferation zones.

## Methods

### Animals

Experiments were conducted using two closely related sympatric species of freshwater crayfish (*Procambarus clarkii *and *Procambarus acutus*) obtained from a commercial supplier (Carolina Biological Supply Company, Burlington, NC). Crayfish were maintained in aquaria with artificial pond water at room temperature and were kept on a 12/12 light/dark cycle. All dissections and manipulations were executed in mid-afternoon, in order to standardize the impact of circadian phenomena.

### In vivo BrdU incubations and detection

In order to monitor the proliferation of cells in the niche, stream and Cluster 10 over time, crayfish were incubated in 5-bromo-2'-deoxyuridine (BrdU; Sigma, St. Louis, MO; No. B5002; 12, 72 or 120 hr; 2 mg/ml pond water) or in a solution of serotonin and BrdU in pond water (18, 24 hr). The dose of serotonin used (10^-9^M) was based on our results from previous studies [18].

Brains of 6 crayfish (carapace length [CL] 11-15 mm) from each group were dissected in cold crayfish saline (205 mM NaCl, 5.4 mM KCl, 34.4 mM CaCl_2_, 1.2 mM MgCl_2 _and 2.4 mM NaHCO_3_) at the specified time points. Brains were fixed in 4% paraformaldehyde in 0.1 M phosphate buffer (PB; 20 mM NaH_2_PO_4_, 80 mM Na_2_HPO_4_; pH 7.4) and processed according to a protocol modified from Zhang et al., 2009 [17]. Briefly, following 2N HCl treatment for 30 min, antibodies (rat anti-BrdU, 1:50; Accurate Chemical, Westbury, NY; No. OBT0030G; mouse anti-glutamine synthetase, 1:100; Becton Dickinson, San Diego, CA; 610517) were applied overnight. Following rinses in 0.1 M PB with 0.3% Triton X-100 (PBTx), brains were incubated in donkey anti-rat IgG Cy2 (1:100; Jackson ImmunoResearch, West Grove, PA; No. 712-225-153) and donkey anti-mouse IgG Cy5 (1:100; Jackson ImmnoResearch; No. 715-175-151) antibodies overnight. The brains were rinsed in PB and mounted in Fluoro-Gel (Electron Microscopy Sciences, Hatfield, PA). In experiments using both BrdU and serotonin incubations, the cell bodies in the niche were labeled with the nuclear stain propidium iodide (PI, 10 μg/ml; 10 min at 18-20°C; Sigma). The PI-labeled profiles were counted, using the GS labeling as a guide for outlining the boundaries of the niche.

### Immunohistochemical localization of serotonin

Standard immunohistochemical methods, as described above, were used to label serotonin in the niche. The following antibodies were utilized: goat anti-5-HT (1:1000; Immunostar, Inc., Hudson, WI), mouse anti-glutamine synthetase (1:100; Becton Dickinson), donkey anti-goat IgG Cy2 and donkey anti-mouse IgG Cy5 (each at 1:100; Jackson ImmunoResearch).

### Pulse-chase BrdU-IdU double nucleoside labeling to determine BrdU clearing time

Groups of crayfish were exposed initially to BrdU (2 mg/ml) for 6 or 24 hr. As prior experiments had ruled out clearing times of less than 12 hours, in the current experiments the crayfish were placed in fresh pondwater for periods of 12 - 102 hr, followed by 6 hr in IdU (2 mg/ml). Brains were removed from the animals at 12-hr intervals and processed and analyzed as in Sullivan et al. [11]. Briefly, BrdU and IdU were detected using a monoclonal rat anti-BrdU (1:50; Accurate Chemical) and monoclonal mouse anti-BrdU (1:50; BD Biosciences Pharmingen, San Jose, CA; No. 347580). Because of the different affinities of these primary antibodies, application of the appropriate secondary antibodies (donkey anti-rat CY2 and donkey anti-mouse CY5; 1:100; Jackson Immunoresearch, West Grove, PA) results in double labeling of cells that have incorporated BrdU and single labeling of cells that incorporate only IdU [11].

### Pulse-chase BrdU-EdU double nucleoside labeling to test self-renewal capacity

Two substitute nucleosides, BrdU and 5-ethynyl-2'-deoxyuridine (EdU), a modified synthetic thymidine analog, were used in a pulse-chase experiment to test whether or not the dividing niche cells are self-renewing. Crayfish (4-6 mm CL) were incubated in BrdU (2 mg/ml in pond water) for 24 hr, then placed in fresh pond water for a duration of either 3.5 days or 7 days, after which they were incubated in EdU (10 μM in crayfish saline) during the last 6 hr prior to sacrifice. We used our standard protocol to process dissected, fixed brains for 20 min in 2 N HCL, followed by PBTx rinses and overnight incubation in rat anti-BrdU (1:50; Accurate Chemical) and mouse anti-glutamine synthetase (1:100; Becton Dickinson). Following PBTx rinses, a fresh preparation of Alexa 488 Click-iT reaction cocktail was applied for 2 hr at 18-20°C. Detection of the incorporated EdU was accomplished by the Click-iT™ reaction, a copper-catalyzed covalent reaction between a fluorophore-coupled azide molecule and an alkyne (in the EdU; Click-iT™ -EdU Alexa Fluor^® ^488 Imaging Kit No. C10337). Secondary antibodies, donkey anti-mouse IgG Cy5 and donkey anti-rat IgG Cy3 (1:100; Invitrogen) followed washes in PBTx. This protocol provided clear images of cells that were either doubly or singly labeled because there is no cross reactivity between the antibodies that bind to BrdU and EdU. Cells with BrdU+ and/or EdU+ labeling in the nucleus, and with glutamine synthetase labeling in the cytoplasm were included in counts of proliferating cells in the niche and streams. Only those cells that doubled-labeled with the nuclear stain propidium iodide and glutamine synthetase, and were located around the vascular cavity, were considered niche cells.

### Niche and dissociated cell co-cultures to test for cell affinities

Dissected and desheathed brains of juvenile crayfish (11-14 mm CL), were placed in culture medium (45 mg/ml bovine serum albumin in crayfish saline plus Gentamicin (50 μg/ml; Sigma, G1397; pH 7.4). Cells from hematopoietic tissue (HPT), hepatopancreas and green gland were dissociated using a protocol modified from [19]. Each tissue type was washed in filtered crayfish saline and then incubated in 250 μl of 0.1% collagenase (Type I) and 0.1% collagenase (Type IV) in filtered crayfish saline for 40 min at 18-20°C. The enzyme buffer was then removed and the tissue washed with 200 μL culture medium, gently passing the tissue through a pipette to separate the cells. After the cells settled, the supernatant was removed and CellTracker™ Green CMFDA (CTG; 10 μM in culture medium; 5-chloromethylfluorescein diacetate; Invitrogen, Carlsbad, CA) was added to the cells for 20 min, followed by centrifugation (800 × g; 4° C; 10 min). The pellet was resuspended in 0.2 ml culture medium. The cell count was calculated with a hemocytometer, and the cell density adjusted to 5.5 × 10 ^5 ^cells/ml. For CTG labeling of hemocytes (blood cells), hemolymph from 3-4 crayfish was immediately added to anticoagulant buffer (NaCl, 0.14 M; EDTA, 10 mM; trisodium citrate, 30 mM; citric acid, 26 mM; glucose, 0.1 M; pH 4.6; [19]) in a 1:2 proportion, then centrifuged, resuspended in saline and incubated in CTG as described above.

The CTG-labeled cell types were introduced into separate Petri dishes containing culture medium with 2-4 dissected brains at a density of at least 5.5 × 10 ^5 ^cells/ml, and distributed by slow circular movement of the dish before placing the co-culture in an incubator for 6 hr at 18°C. Alternative treatment solutions included crayfish culture medium with serotonin at 10^-9^M or methiothepin mesylate salt at 10^-8^M (MMS, a specific *P. clarkii *5-HT_2ß _antagonist [20]; Sigma, M149) into which CTG-labeled hemocytes were introduced, along with dissected desheathed brains. Two other co-culture conditions were tested with CTG-labeled hemocytes: (1) brains which had not been desheathed; and (2) brains from animals treated for 5 days prior to dissection with *p*-chlorophenylalanine (PCPA, 10^-4^M), an irreversible inhibitor of tryptophan hydroxylase [21].

All co-cultures were checked for persistence of the CTG label at the beginning and end of the incubation period. Brains were processed immunocytochemically for mouse anti-glutamine synthetase and propidium iodide, as described above.

### Glutamine synthetase labeling of circulating hemocytes

Hemolymph samples (~100 μl) were drawn from the pericardial sinus of crayfish (11-15 mm CL) with a 1 ml syringe and a 25 3/4 g needle. Hemolymph was spread on slides prepared with 0.01% poly-L-lysine to promote cell adhesion. Hemolymph cells were fixed with 4% paraformaldehyde for 15 min, rinsed for 1 hr with PBTx, and incubated with donkey anti-mouse IgG FAB (Jackson ImmunoResearch; 1:10) and donkey anti-rabbit IgG (Jackson ImmunoResearch; 1:100); samples were fixed again with 4% paraformaldehyde. These treatments were done in order to prevent non-specific binding of subsequent antibodies. Hemolymph smears were then rinsed for another hour, and all but one of the samples from the deprived animals were incubated overnight at 4°C with mouse anti-GS; the slide without this antibody served as a no-primary control to test the specificity of the secondary antibody. Slides were rinsed for 1 hr with PBTx, incubated with donkey anti-mouse IgG CY5 (1:500) for 30 min at room temperature, and rinsed again with PBTx. Finally, the hemolymph smears were incubated with propidium iodide (10 μg/ml) for 5 min at 18-20°C, rinsed with PB and mounted with Fluoro-Gel.

### Dextran injection into the pericardial sinus and vascular cavity labeling

Dextran-fluorescein (MW 3000; 0.5 ml of a 1 mM solution in crayfish saline) was injected into the pericardial sinus of crayfish (20 mm CL). Animals were maintained in pond water for 5 min following injection, during which animals were actively moving around. The brains were then dissected and fixed in 4% paraformaldehyde in PB, processed immunocytochemically for GS, and counter-stained with propidium iodide, as described above. Brains were mounted in Fluoro-Gel and viewed with a confocal microscope.

### Microscopy and counting protocol

Images were acquired on a Leica TCS SP5 confocal microscope equipped with argon 488 nm, and 561 and 633 nm diode lasers. Serial optical sections were taken at intervals of 1 μm and saved as both three-dimensional stacks and two-dimensional projections. Cell profiles were counted by tracing projections of the cells onto transparent sheets attached to the screen of the confocal monitor. Images of control and experimental animals were randomized, and cell counting was carried out blind. For blood smear analysis, four 5-10 μm confocal stacks were taken from non-overlapping regions on one slide. Image preparation and assembly were done in Adobe Photoshop 7 (Adobe Systems, San Jose, CA). Adjustments were made only to the color balance and contrast of the images.

### Statistics

Comparisons among different treatments of animals were carried out with one-way ANOVA analysis followed by Tukey's multiple comparison test using SPSS (IBM), unless otherwise indicated. Chi-square analysis (SPSS) was used to determine the significance of cell-type affinity in co-cultures.

### RT-PCR

Total RNA was extracted from crayfish tissues with TRI_ZOL _reagent according to the manufacturer's instructions (Invitrogen). Reverse transcription was performed following the RNA extraction, and first-strand cDNA was synthesized with a random hexamer and SuperScript III reverse transcriptase (Invitrogen). PCR amplification of cDNA was performed using REDTaq ready mix PCR reaction mix (Sigma). The forward and reverse primers of 5-HT_1α _and 5-HT_2β _were designed based on published sequences for *P. clarkii *(GenBank accession numbers EU131667 and EU131666). They are 5**'**-AGAACACGACGAGCGATGA-3**' **and 5**'**-GCCAAGAATGACGGAAGTAA-3**' **(5-HT_1α_) and 5**'**-GATCTGTCCGCTGGAAGAAG-3**' **and 5**'**-ACCTGAAGCTCGAGTCGTGT-3**' **(5-HT_2β_). In the negative control, ddH_2_O was used instead of the cDNA template.

### Western Blot

Brain samples were lysed with RIPA lysis buffer (50 mM Tris pH 7.5, 150 mM NaCl, 1% Triton X-100, 1% Nadeoxycholate, 0.1% SDS and Halt proteinase inhibitor [Pierce, USA; No. 78430]). Total protein concentration was determined by the bicinchoninic acid (BCA) protein assay method (Pierce; No. 23227). Protein samples (40 μg total protein in each sample) separated by 10% SDS-PAGE were electroblotted onto an Immuno-PSQ PVDF membrane (0.2 μm, Millipore). The membrane was blocked with 4% skimmed milk in TBST buffer (10 mM Tris pH 8.0, 150 mM NaCl, 0.1% Triton X-100) for 1 hr at room temperature. Incubation with 5-HT_1α _or 5-HT_2β _antibody (1:500 and 1:10,000 respectively, gift from D. Baro, Georgia State University, Atlanta, GA) was typically done for 16 hr at 4°C on a rocking platform. HRP-coupled goat anti-rabbit IgG antibody (Jackson ImmunoResearch; No. 111-035-003) was added and incubated at 24°C for 1 hr. After washing, signals were developed by reaction with WESTSAVEup (AbFrontier, Korea) and visualized according to the manufacturer's instructions. To re-probe for actin in the samples on the same membrane, the initial probed antibodies were removed from the PVDF membrane by submerging the membrane in stripping buffer (2% SDS, 100 mM β-mercaptoethanol, 62.5 mM Tris-HCl pH 6.7) at 50°C for 30 min with occasional agitation. The PVDF membrane was then washed in TBST, blocked and reprobed sequentially with mouse actin (Chemicon, USA; MAB1501; 1:3000) and goat-anti-mouse IgG HRP (Jackson ImmunoResearch; No. 115-035-003). Signals were developed as described above.

## Results

### I. Testing the Self-renewal Capacity of Crayfish Neural Stem Cells

Our previous morphological studies indicated that the 1^st^-generation neuronal precursor cells residing in the neurogenic niche in the crayfish brain undergo only geometrically symmetrical divisions, and that both daughter cells migrate from the niche towards the proliferation zones [17], suggesting that the niche precursors are not self-renewing. In the proliferation zones, the 2^nd^-generation cells divide at least once more and their descendants differentiate into interneurons innervating the primary olfactory regions (the olfactory lobes) and higher-order processing areas (the accessory lobes) [11,15]. One goal of the current experiments was to directly test the self-renewal capacity of the 1^st^-generation neuronal precursors in the adult crayfish brain, to be certain that asymmetric divisions and retention of daughter cells in the niche were not overlooked in prior studies.

#### Long BrdU incubations and niche cell cycle time

**Figure 2:**
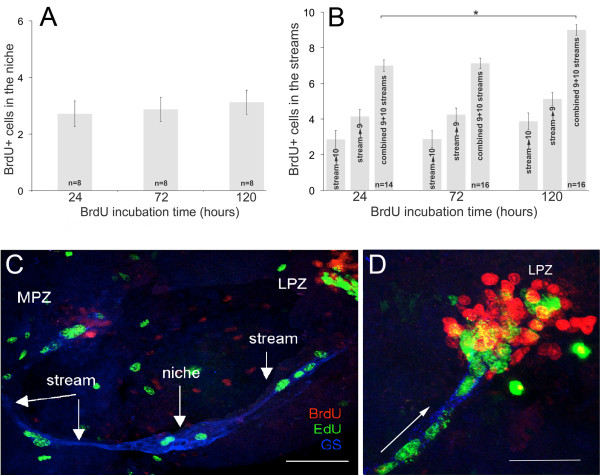
**Experiments reveal the cell cycle time and lack of self-renewal capacity of the 1**^**st **^**generation neuronal precursors**. **A**. Graph of average numbers of BrdU-labeled cells in the niche after BrdU incubation for 24, 72 or 120 hours, followed by immediate sacrifice. The mean number of BrdU-labeled cells is between 2 and 3 for all groups, and there is no statistical difference between groups. **B**. Graph of average numbers of BrdU-labeled cells in the streams after BrdU incubation times of 24, 72 and 120 hrs. Mean counts are shown for the streams between the niche and Cluster 9, between the niche and Cluster 10, and the combined counts in the streams. **C**. Live crayfish were incubated in BrdU for 24 hours and then maintained in fresh pond water for 7 days. Just before sacrifice, they were treated with EdU (green) for 6 hours. Fixed brains also were labeled immunocytochemically for glutamine synthetase (blue) to reveal the niche and streams (C, D). S phase cells in the niche and streams are labeled only with EdU, demonstrating that migration is uni-directional (away from the niche) and that niche cell divisions are not self-renewing. **D**. Higher magnification image of the LPZ. The BrdU-labeled cells, which were labeled first in the sequence, are found only in the proliferation zones (MPZ and LPZ) and not in the niche. Arrow indicates direction of migration. Scale bars: C, 100 μm; D, 50 μm.

If the niche cells are self-renewing, increasingly long incubation times in BrdU followed by immediate sacrifice might be expected to label increasing numbers of cells in the niche, as observed in crustacean embryos where neuroblasts are surrounded by their progeny [22,23]. However, regardless of whether crayfish are incubated in BrdU for 24, 72 or 120 hr (Figure 2A, C) we observe only 2-4 BrdU-labeled cells in the niche, 1-2 on either side of the vascular cavity. The migratory streams, which are the only egress from the niche, do contain more BrdU-labeled cells in the 120-hr than the 24-hr preparations (2-tailed t-test, p < 0.05; Figure 2B), as might be expected if the speed of migration is slow relative to the niche cell cycle time. However, in no case did we find more than 10 labeled cells, with gaps between them, spaced along the streams (Figure 2C). The small number of BrdU-labeled migrating cells, even after long BrdU incubation times (and the long migration time of 5 to 7 days [11]), excludes the possibility that the niche cells cycle rapidly. If these 1^st^-generation niche precursor cells have a cycle time of less than 24 hr, then one would expect to see much higher numbers of cells in the streams and a greater difference in the numbers between the 24-hr and 120-hr BrdU-exposure times. Further, a few migrating cells are in the cell cycle and proceed through mitosis during migration [17], as do cells in the rostral migratory stream [24]. The degree of BrdU incorporation during migration along the streams can be expected to be similar in all groups (24, 72 and 120 hr), and so differences between the counts at shorter (i.e., 24 or 72 hr) vs. longer (120 hr) BrdU exposures (but shorter than the migration time) represent the output of the niche, from which cycle time can be deduced. Using this approach, we infer a *minimum *cell cycle time of 48 hr for the niche precursor cells. Consistent with this result, we find no change in the number of cells in the streams between 24 and 72 hr (Figure 2B).

Another approach to estimating cycle time is to consider that the migratory streams are, essentially, a sink receiving the cells produced by the niche. BrdU cell labeling should reach a plateau at the maximal migration time (~120 hr), reflecting the output of the niche precursor cells over that period. Indeed, the maximum number of BrdU-labeled cells we have observed along the total length of the streams in the present study is 10 (Figure 2B), including cells that may have incorporated the BrdU during migration. Dividing by the ~5-day migration time therefore also suggests a *maximal *niche output of ~2 cells/day. Given that there are generally two cells in S-phase in the niche at any specific time (one on each side of the niche) and making the assumption that both daughter cells migrate away from the niche [17], this suggests that each niche precursor cell divides *no more than *once every 48 hr. Because a few cells in the streams will have arisen from mitoses during migration [17], a 48-hr cycle time is therefore a conservative estimate of the length of the cell cycle in the 1^st^-generation niche precursors.

#### BrdU clearing time and double-nucleoside labeling

In order to further test the self-renewal capacity of the niche precursor cells, double-nucleoside labeling experiments were conducted. In order to design these experiments, a knowledge of the BrdU clearing time was necessary, and this was defined using a BrdU-IdU pulse-chase approach. Two groups of crayfish were incubated in BrdU for either 6 hr or 24 hr and then returned to fresh pond water. Crayfish were then sacrificed at 12-hr intervals after the end of the BrdU incubation; these crayfish were exposed to IdU during the last 6 hr prior to sacrifice. In order to determine the BrdU clearing time, the crayfish brains were examined for the first appearance of cells that were labeled solely for IdU. This occurred 30-36 hours for the crayfish that were incubated in BrdU for 6 hr, and 42-48 hr for those that were incubated in BrdU for 24 hr, in what appeared to be glial cells near cell cluster 6 in the brain. The clearing time for the BrdU therefore is slightly longer for longer exposures to the nucleoside. Even more important, however, is the finding that the clearing time for BrdU in crayfish is much longer (>30 hr) than in mammals, where this is estimated to be as short as 2 hr [25].

To test the self-renewal capacity of the niche precursor cells, pulse-chase BrdU-EdU studies were then conducted. Animals were maintained in pond water for 3.5 or 7 days between the BrdU (24 hr) and EdU (6 hr) exposures, and sacrificed immediately after the EdU incubation. Based on the clearing time analysis, BrdU would have persisted for 42-48 hr after the BrdU was removed, or through roughly half of the 3.5 day maintenance period in pond water. These experiments result in only EdU-labeled cells in the niche; all BrdU labeling is confined to the streams (3.5 days) and/or proliferation zones (3.5 and 7 days) (Figure 2C, D). These data demonstrate that the earlier-labeled (BrdU) group of cells has migrated away from the niche, and that none are left behind to divide and maintain the niche population, consistent with our earlier findings [17]. These studies also confirmed the migration time (5-7 days, [11]) along the streams, because many BrdU-labeled cells in the 3.5 day group had not yet reached the proliferation zones.

### II. Niche Cell Numbers Can Increase Without Niche Cell Divisions

**Figure 3:**
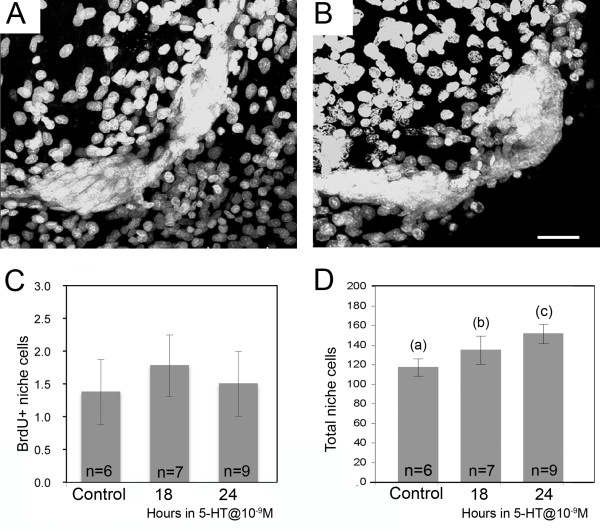
**In vivo exposure to serotonin increases total niche cell numbers, but does not alter the number of dividing niche cells**. **A**. Images of niches stained with propidium iodide from control (A) and 24-hr 10^-9 ^M serotonin-treated (B) crayfish. **C**. Graph of mean numbers of BrdU+ cells per niche in controls and those exposed to 10^-9 ^M serotonin for 18 and 24 hours. 5-HT does not alter the number of BrdU-labeled cells in the controls (ANOVA: p = 0.960). The number of BrdU-labeled niche cells is lower in this experiment than the one graphed in Figure 2A because these crayfish were slightly larger/older; the cell cycle of niche precursors slows as the animals age [17]. **D**. However, there are significant increases in the number of propidium iodide stained cells in the niche in 5-HT-treated crayfish compared with untreated controls (ANOVA: p < 0.0001 followed by Tukey multiple comparisons). Different letters above the histograms indicate significant differences among the groups. Error bars = standard error of the means. n = numbers of niches per group. Scale bar refers to A and B, 50 μm.

Incubation of animals in 10^-9 ^M serotonin, which is known to stimulate neurogenesis in these animals [18,26-29], increases the number of BrdU-labeled cells in the Cluster 10 proliferation zones but does not alter the number of S phase (BrdU-labeled) cells in the niche (Figure 3C) or streams [29]. However, the total number of cells in the niche in serotonin-treated animals increases by >20% in 24 hr (Figure 3). This highly significant increase (ANOVA, p < 0.0001, followed by a Tukey-Kramer HSD) in the total number of niche cells in the absence of an increase in the number of S phase cells, may also suggest that the 1^st^-generation neuronal precursor cells in the crayfish niche are not self-renewing because their numbers can increase without additional cell divisions among the niche cells. The underlying assumption here, however, is that all of the niche cells have the potential to become neuronal precursors, an idea that is supported by four lines of previously published evidence (see Discussion).

### III. Possible Extrinsic Sources of Neuronal Stem Cells

To explore possible relationships between the niche and other tissues, several cell types (green gland, hepatopancreas, hematopoietic tissue, hemocytes) were isolated from their respective tissues and labeled with the fluorescent marker CellTracker™ Green CMFDA (CTG; Invitrogen). Labeled cells were then introduced into culture dishes containing freshly dissected, desheathed crayfish brains, followed by a 6-hr incubation period at 18°C. The distribution of labeled cells in each culture dish was subsequently visualized to determine whether cells showed any affinity for the brains and/or associated niches.

**Figure 4:**
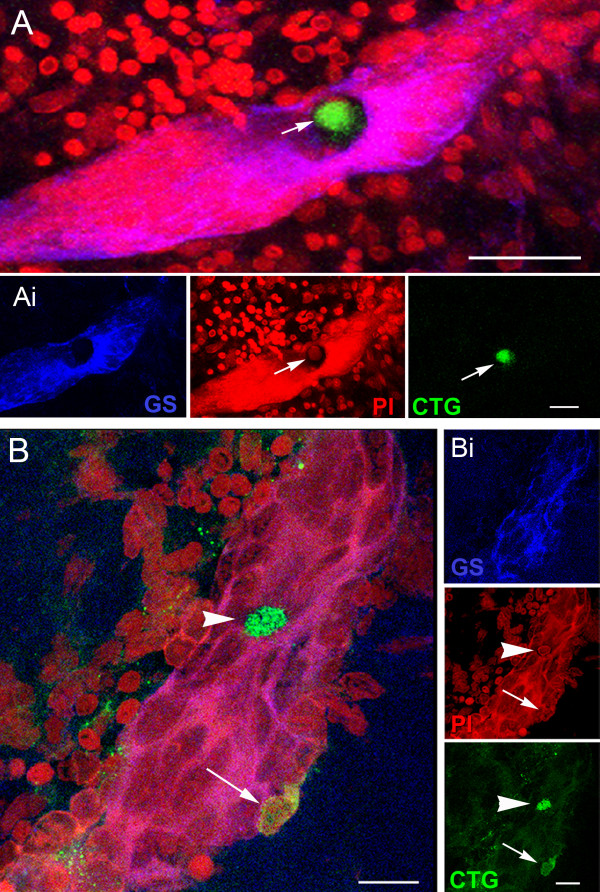
**CellTracker™-labeled cells are found in the vascular cavity and in the neurogenic niche**. **A-B**. Projections of confocal stacked images of neurogenic niches. **A**. Position of a CTG-labeled cell in the vascular cavity with all fluorescence channels merged. **Ai**. Glutamine synthetase (GS, left) labeling of the niche; propidium iodide (PI, center) labeling of all cell nuclei, revealing the niche cell cluster with arrow pointing to the nucleus of the CTG-labeled cell; CTG-labeled cell (right). **B**. In another co-cultured brain, a CTG-labeled cell (arrowhead) resides in the cavity and a second CTG-labeled cell (arrow) is embedded in the outer edge of the niche. **Bi**. Top, GS labeling of the niche; Middle, PI labeling of cell nuclei with arrowhead and arrow pointing to the corresponding nuclei with CTG-labeling in B; Bottom, CTG-labeled cells. Scale bars: A, 50 μm; Ai, 20 μm; B and Bi, 20 μm.

**Figure 5:**
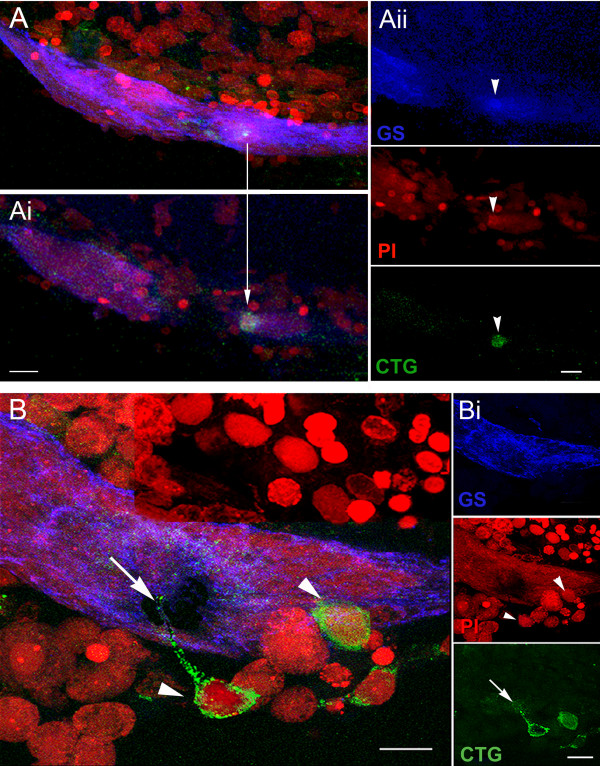
**Some CellTracker™-labeled cells in the niche are also immunoreactive for glutamine synthetase**. **A**. Projections of stacked images from the most ventral region of the neurogenic niche reveal a CTG-labeled cell just below its surface. **Ai**. Confocal scans taken deeper in the niche expose the CTG-labeled cell. An arrow drawn between **A **and **Ai **show the same CTG-labeled cell at different depths within the niche. **Aii **are the separate channels for GS labeling in the niche (top); PI revealing cell nuclei (middle); and CTG-labeled cell (bottom). **B**. Other CTG-labeled cells, although not labeled with glutamine synthetase (GS), have interesting morphological characteristics. In B there are labeled cells just outside the niche (arrowheads), one with a long process extending into the niche (arrow), and another CTG-labeled cell that appears to be inserting into the outer margin of the niche. **Bi**. Top, middle bottom are separate channels: GS labeling the niche (top); PI revealing cell nuclei, arrowheads pointing to the respective CTG-labeled cells in B (middle); and CTG-labeled cells with arrow pointing to the fine process coming from one of these (bottom). Scale bars: A, Ai, Aii and B, 20 μm; Bi, 10 μm.

Of the various cell types tested, most remained evenly distributed in the culture dishes and showed no particular attraction to the brains or niches (Table 1). For instance, in brains incubated with CTG-labeled cells from hepatopancreas, 10 out of 11 niches were devoid of labeled cells in the vascular cavity and niche. In contrast, cells extracted from the hemolymph showed a remarkable affinity for the niche. In 77% of niches co-cultured with CTG-labeled circulating hemocytes, these cells were found in the vascular cavity of the niches (Figure 4A) and among the precursor cells in the niches (Figures 4B, 5); some of these cells also labeled for glutamine synthetase (Figure 5A), a marker of the 1^st^-generation niche precursor cells in these crayfish (Figure 1A, C).

**Table 1:** Hemocytes are attracted to the neurogenic niche.

**1**. Dissociated cells from crayfish tissues labeled with CTG.	Total number of niches	Percent of niches with CTG labeled cells
Green Gland	10	0%

Hepatopancreas	11	9%

Hematopoietic Tissue	12	9%

Hemocytes	30	77%

**2**. Changes in cell culture medium with CTG labeled hemocytes		

Hemocytes + 5-HT × 10^-9 ^M	12	17%

Hemocytes + MMS × 10^-8 ^M	16	31%

**3**. Treatment prior to co-culture, culture + CTG labeled hemocytes		

5-day PCPA pretreatment	8	12.5%

Sheath left on dissected brain	12	0%

The relationship between the neurogenic niche and dissociated CellTracker™ Green (CTG)-labeled cells from four crayfish tissues (green gland, hepatopancreas, hematopoietic tissue and hemolymph) was examined in co-cultured, desheathed brains. Complete exposure to the niche/ventral brain surface appears to be essential for the niche region to attract CGT-labeled hemocytes in vitro, because no cells are attracted to the niche when sheathed brains are placed in co-culture with CTG-labeled hemocytes. The number and the percentage of total niches containing CTG+ cells appear in the second and third columns. The Chi-square test of independence was used to test the significance of these findings; the result is chi square (7, *N *= 111) = 34.8885, p < 0.001, indicating that the number of niches containing CTG+ cells was significantly dependent on the cell type. The attraction of hemocytes to the niche relative to other cell types is therefore of statistical significance.

### IV. Serotonin and the Neurogenic Niche

**Figure 6:**
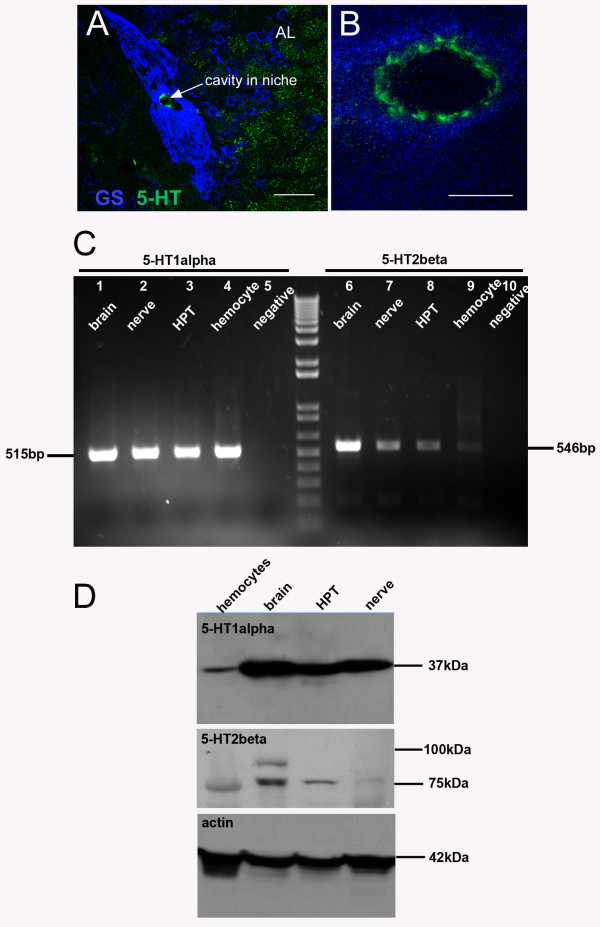
**Serotonin is localized in the neurogenic niche and serotonin receptor subtypes are expressed in hemocytes and brain**. **A**. The neurogenic niche labels immunohistochemically for glutamine synthetase (blue) and serotonin (green). The vascular cavity is outlined by a rim of serotonin labeling, clearly seen in a higher magnification image shown in B. The additional serotonin labeling observed in (A) near the niche is localized in terminals in the accessory lobe. **C**. RT-PCR reveals the expression of 5-HT_1α _(left) and 5-HT_2β _(right) receptor subtype mRNAs in brain (lanes 1, 6), nerve (lanes 2, 7), hematopoietic tissue (HPT, lanes 3, 8), hemocytes (lanes 4, 9) and the negative control (lanes 5, 10). **D**. Western blot for 5-HT_1α _(top) and 5-HT_2β _(middle) receptor proteins, with actin control (bottom). Scale bars: A, 50 μm; B, 10 μm.

Our studies (e.g., Figure 3) suggest that serotonin may serve as a signal to attract cells to the niche. To explore this possibility, immunocytochemical techniques were used to ask whether the niche contains serotonin. Indeed, serotonin immunoreactivity is found in a discontinuous ring at the rim of the vascular cavity (Figure 6A, B). How this structure may be associated with the niche precursor cells is not known, as none of the niche cells were themselves labeled. The cellular origins of this structure are not known.

Because increased serotonin levels expand the niche precursor cell population in vivo and because serotonin is localized around the vascular cavity, we further explored the relationship between circulating cells and the niche by introducing serotonin or the *P. clarkii*-specific 5-HT_2ß _receptor antagonist methiothepin mesylate salt (MMS) into the brain-hemocyte co-cultures. Our hypothesis was that if serotonergic mechanisms are involved in the attraction between hemocytes and the niche in our co-cultures, then serotonin or molecules that would interfere with serotonergic mechanisms might alter the behavior of the cell type responsible for increases in the number of niche cells. Indeed, in brain-hemocyte co-cultures to which 10^-9 ^M serotonin was added, labeled cells were found in only 17% of the niches/vascular cavities, a significantly lower rate of cell incorporation into the niche than brain-niche co-cultures incubated in normal medium without serotonin (Table 1). Likewise, in brain-hemocyte co-cultures to which 10^-8 ^M of the *P. clarkii*-specific 5-HT_2β _receptor antagonist MMS [20] was added to the normal culture medium, CTG-labeled cells were found in only 5 of the 16 vascular cavities, and no CTG-labeled cells were found in the niche cell clusters (Table 1). These results demonstrate a change in cell behavior as a result of exposure to serotonin or MMS, suggesting that serotonergic signaling mechanisms may at least partly explain the affinity between the CTG-labeled hemocytes and the niche. This conclusion is also supported by experiments where crayfish were exposed to PCPA for 5 days to decrease serotonin synthesis, prior to dissecting the brains for co-cultures. In these preparations, attraction of hemocytes to the niche was also severely reduced (Table 1).

We also hypothesized that if serotonin is acting directly to attract cells to the niche, some circulating hemocytes should contain serotonin receptors. RT-PCR shows that mRNAs of serotonin receptor subtypes 1α and 2β are present not only in the nervous system, but also in hematopoietic tissue (HPT) and hemocytes (Figure 6C). In Western blots, antibodies against crustacean 5-HT_1α _and 5-HT_2β _receptors label proteins around 37 kDa and 80 kDa, respectively (Figure 6D).

### V. Glutamine Synthetase Immunoreactivity: A Marker of Neuronal Precursor Cells and a Subtype of Hemocytes

**Figure 7:**
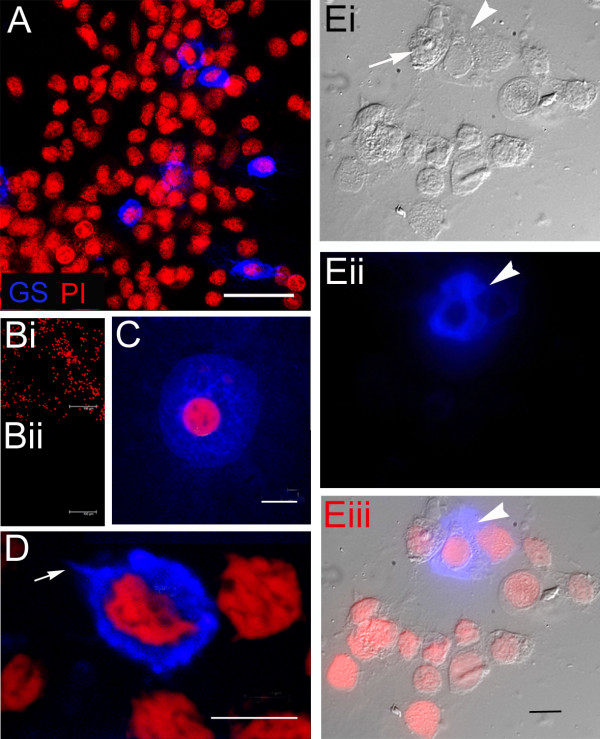
**Hemocytes from crayfish were labeled immunocytochemically for glutamine synthetase**. **A**. GS+ cells (blue) were found in a small percentage (1-3%) of crayfish hemocyte samples (n = 24 smears from 12 crayfish). **B**. A no-primary control showed that the antibody was specific for GS (no non-specific labeling in the blue channel, Bii), although many cells were labeled with propidium iodide (Bi). **C, D**. Higher magnification images reveal some cells that have a high cytoplasmic to nuclear ratio; other GS+ cells had one or more pseudopodia (D, arrow). There are three categories of circulating cells in crayfish based on morphological criteria [31], two of which can be distinguished in Ei. The image in Ei shows the outline of the cytoplasm and nucleus of a GS+ cell (arrowhead), which appears to be of the semi-granular variety. A large granular hemocyte (white arrow) with large refractive granules is also in the field of view. The fluorescent channel alone (Eii, arrowhead) and Eiii (composite: Ei plus Eii) reveal one labeled GS+ hemocyte. Hemocytes were counter-stained with propidium iodide (red) and were imaged using confocal microscopy (A-D) and Nomarski optics (Ei, Eiii). Scale bars: A, 50 μm; B, 100 μm; others, 10 μm.

Among the CTG-labeled cells extracted from the circulation that were attracted to the niche, are cells that show immunoreactivity to glutamine synthetase (e.g., Figure 5A), which is also a marker of the 1^st^-generation neuronal precursors in the procambarid neurogenic niche (see Figure 1A, C). We therefore asked whether any cells circulating in the hemolymph label for this marker. Indeed, one type of circulating cell labels immunocytochemically with GS antibodies (Figure 7), a finding that has been confirmed by Western blot analysis [14]. The GS-immunoreactive cells have fine cytoplasmic granules and often have pseudopodia and/or processes (e.g., Figure 7D), consistent with the phenotype of semi-granular hemocytes [30,31].

## Discussion

### Self-renewal of neural stem cells

Numerous questions punctuate any discussion of adult neurogenesis. Central to these issues is the idea that the primary, 1^st^-generation neuronal precursors are capable of long-term self-renewal and of generating lineages that produce neuronal and glial cells. In mammals, the self-renewal ability of stem cells was largely established in vitro via neurosphere and adherent monolayer cultures [10]. Suh et al. [9] provided the first in vivo evidence of stem cell properties of hippocampal neural progenitors in the adult mammalian brain, where type 2 Sox2-positive cells were shown to self-renew and give rise to both neurons and astrocytes. The question of self-renewal of primary adult neuronal precursors in non-vertebrates is of interest because of the paucity of in vivo data from vertebrate systems and for evolutionary reasons. Because adult neurogenesis is found in a diverse group of vertebrate and invertebrate species, it is crucial to understand the properties of neuronal precursors across this range, and to recognize shared features that persist in the face of evolutionary pressures. Evolutionary conservation and/or convergence, are frequent features of fundamental biological mechanisms (e.g., ionic basis of the action potential [squid axon, [32]], mechanisms of learning [Aplysia californica, [33]] and cell death [C. elegans, [34]]). Indeed, many characteristics of adult neurogenesis in crayfish are strikingly convergent with those in mammals, such as the presence of a vascularized niche that supports the progenitor cells, which serve as both precursor and support cells, and the directed migration of intermediate precursor cells. While these features may or may not represent the conservation of an early mechanism shared by a common ancestor of crayfish and mammals, the parallels are close. Common strategies may well emerge from the study of such models (such as the possible tissues from which neuronal precursor cells can be derived), that have potential implications for phylogenetically more advanced organisms.

A characteristic feature of neurogenic niches in vertebrate and invertebrate organisms is vascularization [35]. In the decapod crustacean brain, the 1^st^-generation neuronal precursors reside in a niche that has many of the same properties as stem cell niches in vertebrate and non-vertebrate systems [11,16]. The crayfish niche lies on top of a blood vessel; communication with the circulation via a vascular cavity has been demonstrated by injecting fluorophores conjugated to dextran into the pericardial sinus and allowing the heart to distribute the dye (Figure 1B), or by injection into the dorsal artery that perfuses the midbrain [11].

There has been debate surrounding the properties of the primary neuronal precursors in the decapods, with some arguing in favor of a neuroblast that persisted after embryogenesis, residing among supporting niche cells, as a source of adult-born neurons [36,37]; this hypothesis states that large, tetraconate-typical neuroblasts act as neuronal stem cells in both *Panulirus argus *and *P. clarkii*, and that these undergo rapid, self-renewing divisions [37]. These studies have shown the presence of a large nucleus that differs significantly in size and shape from the nuclei of other cells in the neurogenic niche, and which has therefore been identified as a putative neuroblast. However, functional or developmental data in support of the neuroblast hypothesis, or evidence of asymmetric divisions, are lacking. Further, our histological and electron microscopic studies in *P. clarkii *have not revealed consistently-placed distinctive, large cells in the niche cell cluster [[17]; Allodi and Beltz, unpublished results]. In contrast, we have argued that adult neurogenesis in the decapods does not depend on the function of single, persistent neuroblasts but rather on a population of cells that appear to act as both precursor and support cells [11]. This hypothesis is based in part on the finding that all niche cells in *P. clarkii*, including those that are mitotic, label with an antibody against glutamine synthetase, suggesting a relationship among all the niche cells. Further, labeling of all niche cells with an antibody against the G1-phase marker MCM2-7 (except when they are progressing through S to M phase), suggests that these are not terminally differentiated cells as one would expect if some niche cells are serving in a support role for other, actively dividing neuronal precursors. In addition, niche precursor cells undergo geometrically symmetrical divisions and both daughters appear to exit the niche (Figure 1C) [17], a feature that is uncharacteristic of neuroblasts. Finally, the fact that animals living in enriched conditions have significantly higher numbers of primary neuronal precursor cells actively in S-phase (BrdU-labeled) in the niche relative to animals reared in deprived conditions [38] confirms that the neuronal precursor cells are not uniquely endowed neuroblasts, but rather comprise a population of cells whose status in the cell cycle can be adjusted in response to local conditions. The mode of division of the niche precursors also caused us to focus our attention on the question of self-renewal among these cells. The primary goal of the present study, therefore, was to directly test the self-renewal capacity of these primary neuronal precursor cells.

Our current data indicate that the 1^st^-generation neuronal precursor cells in the crayfish brain are *not *self-renewing and, therefore, that there must be a source extrinsic to the niche that is capable of replenishing this population of cells: (i) If the niche cells are self-renewing, increasing the incubation time in the S-phase marker BrdU might be expected to label increasing numbers of cells in the niche. However, regardless of BrdU incubation time, we observe the same number (2-4) of BrdU-labeled cells in the niche (Figure 2A). (ii) Pulse-chase BrdU-EdU experiments, where animals were maintained in pond water for several days between BrdU and later EdU treatments, result in only EdU-labeled cells in the niche; BrdU labeling is confined to cells in the migratory streams and/or proliferation zones (Figure 2C, D). Our interpretation of this result is that the earlier BrdU-labeled cohort of cells has migrated away from the niche, and that none are left behind to maintain the niche population. It is highly unlikely that the BrdU label in these cells could have been diluted below detection, because the cycle time of the niche precursors (>48 hr) and the BrdU clearing time (36-42 hr) are relatively long compared to the length of the chase period (3.5 or 7 days).

A direct test of the niche cell cycle time has been complicated by the small pool of actively cycling niche cells and their apparently rapid departure from the niche. It is known that the cycle time of the niche cells decreases as the animals grow and age [17]. In small crayfish (<15 mm CL), BrdU labeling over periods of <4 hr does not reliably label niche cells, and labeling that does occur is very weak; issues related to BrdU uptake as well as speed of progress in the S phase to incorporate a detectable level of BrdU may contribute to this lag time. BrdU labeling for 6 or more hours invariably labels 2-4 niche cells, 1-2 on either side of the vascular cavity. When two cells are labeled, each of these is half the size of a singly labeled cell. Our interpretation of this observation is that the pairs of labeled cells represent cells that have undergone M phase during the BrdU incubation, and that unpaired, large cells have not yet reached M phase. Only on rare occasions have there been >4 BrdU labeled cells observed in a niche in animals of any size that are maintained in control conditions.

"Steady-state" in the crayfish niche is, therefore, 2-4 cells (see Figure 2A). As more than 4 cells rarely accumulate in the niche in crayfish exposed to control conditions, the usual method for determining the length of the cell cycle (T_C_) by defining the percentage of cells relative to steady state that incorporate BrdU during incremental increases in BrdU exposure time (e.g., 1 hr, 2 hr, 3 hr, 4 hr) [39], is not applicable. Such a graph changes sharply from 0% to 100% (3-4 cells) within 6 hr for small crayfish (<15 mm CL). Constant exposure to BrdU for 8 hr [17], 10 hr [11], 24, 72 or 120 hr (Figure 2) or for two weeks [11] yields precisely the same result.

For these reasons, we infer the cycle time of the niche cells by counting BrdU-labeled cells in the migratory streams, which are the only conduit by which cells leave the niche. We reason that if the niche cells have a short cycle time, then increasing incubation times in BrdU over 1-5 days should lead to large increases in BrdU-labeled cells in the streams, as the transit time from the niche to the Cluster 9 and 10 proliferation zones (MPZ and LPZ, respectively) is 5-7 days [11], a time that was confirmed in the current set of experiments. However, regardless of whether the animals were exposed to BrdU for 24, 72 or 120 hours (Figure 2B), the streams always contained fewer than 10 cells. The long migration time for the 2^nd^-generation precursors and the stability of their numbers even following a long BrdU exposure (e.g., 120 hr), suggests a slow cycle time for the 1^st^-generation niche precursor cells [>48 hr].

A final possibility that could account for our BrdU-EdU results, is if chromosomes segregate non-randomly during divisions, so that the older "parental" DNA is retained in daughter stem cells, while the new strands are relegated to the daughter that will differentiate. This immortal strand hypothesis proposed by Cairns in 1975 [40] predicts that the stem cell DNA will not be labeled with pulsed nucleotide analogs after their first division; hence these markers would be ineffective in revealing the 1^st^-generation neuronal precursors. We believe this situation does not apply to the crayfish niche because of the numbers of mitotic cells that we have observed; in all cases these have been geometrically symmetrical divisions and segregation of BrdU-labeled DNA into both daughters was observed in telophase cells. While our experiments cannot completely rule out the possibility that niche cells transition sporadically to a self-renewing mode (e.g., [41]), this phenomenon would have to be a common event in order to maintain the niche cell population.

### A source of neuronal stem cells?

Based on these data indicating that the 1^st^-generation neuronal precursor cells in crayfish are not self-renewing, we asked what tissues might replenish the supply of neuronal precursors in the brains of these animals. An external source is necessary in order to maintain niche cell numbers, so that the niche is not depleted of precursor cells as these divide and the daughters migrate away. We have tested a variety of cell types that were labeled with CellTracker™ Green, using in vitro co-cultures with dissected brains. In these studies, we asked whether any of these cell types show an affinity for the brain and/or niche. The only cell type that was consistently associated with the niches in these co-cultures were cells extracted from the circulation (Figures. 4, 5; Table 1). The specific identity(ies) of these hemocytes has not been determined, although the granular cytological features and processes observed in many of these suggest they may be of the semi-granular variety that have been proposed by some to be hematopoietic stem cells in crustaceans [30,31]. Co-cultures created with dissected brains and CTG-labeled cells from a variety of other tissues do not show the attraction for the niche observed with labeled hemocytes (Table 1).

Incubation of live crayfish in serotonin for 18-24 hr increases the number of niche cells (Figure 3A, B, D), without any change in their cell cycle time (BrdU labeling). This indicates that a source extrinsic to the niche must have the capacity to replenish the niche cell population, and that serotonin can influence this process. We therefore performed additional co-culture experiments with serotonin or the *P. clarkii*-specific 5-HT_2β _receptor antagonist MMS to test whether hemocyte affinity for the niche was influenced by serotonergic mechanisms. Indeed, these treatments significantly reduced the number of cells found in the vascular cavity and niche. We hypothesize that the cultured brains/niches may be a source of serotonin that attracts the hemocytes, and that the presence of serotonin in the culture may obliterate or mask any signaling gradient. Further, the finding that MMS severely reduces the ability of CTG-labeled cells to invade the vascular cavity, may indicate that the attraction of labeled cells to the niche is mediated at least in part by 5-HT_2ß _receptors. In support of this serotonin hypothesis, a discontinuous ring of serotonin immunoreactivity is found at the rim of the vascular cavity (Figure 6A). The cellular source of this serotonergic labeling is not known, as the niche cells themselves do not label; this issue is currently under investigation. We anticipate, however, that serotonin's influence is necessary but not sufficient, because other serotonergic structures in the brain do not attract hemocytes, suggesting that additional factors, presently unidentified, are likely to be important in the affinity between hemocytes and the niche. Further, we presume that the molecular machinery underlying the attraction of hemocytes for the niche must be acquired during the process of hemocyte maturation and their release from the hematopoietic tissues, as the cells dissociated from hematopoietic tissues show little affinity for the niche in our co-cultures.

There are additional reasons for suspecting a close relationship between the hematopoietic system and the niche in crayfish: (1) The crayfish niche is a discrete organ that is physically isolated from other tissues with the exception of the circulatory system, with which the niche has contact through the vascular cavity (e.g., Figure 1B) and the extracellular milieu, as there appears to be no membrane enclosing the niche. The migratory streams connecting the niche with the proliferation zones have been shown to support migration only away from the niche [11], and it is therefore unlikely that they participate in the maintenance or renewal of the niche cell population. We therefore propose that, by way of the vascular connection, the hematopoietic system has the *opportunity*to interact with the niche via its production of circulating cells. (2) A small population of hemocytes labels immunocytochemically for glutamine synthetase (GS), a marker of the niche stem cells (Figure 7) (see also [14]). Further, the proportion of GS-immunoreactive hemocytes increases after 2-3 weeks of environmental enrichment, a treatment that also promotes neurogenesis in these animals [38]. (3) Hemocytes express 5-HT_1α _and 5-HT_2ß _receptors (Figure 6C, D). Cells that expand the niche pool in response to serotonin would be expected to express these receptors, if the serotonergic effect is a direct one. The finding that addition of serotonin or MMS to the culture alters the affinity of vascular cells for the niche is also consistent with the presence of these receptors on hemocytes. (4) Niche precursor cells have the same large dimensions and unusual chromatin pattern that are seen in some circulating and perivascular cells that are thought to be of hematopoietic origin, and indeed semi-thin sections show one cell type (Type III) that has a stalk connecting the cell body to the niche [17]. This feature is highly reminiscent of CTG-labeled hemocytes that insert into the niche (e.g., Figure 5B), although the semi-thin sections in our previous studies were prepared from freshly dissected and fixed brains from untreated crayfish.

### Significance of these findings

This work provides an example of 1^st^-generation neuronal precursor cells that apparently do *not *undergo self-renewing divisions, indicating that these neuronal precursors in adult crayfish must be replenished from a source external to the neurogenic niche. Our experiments also show that in co-cultures, hemocytes have an affinity for the niche, and that some of these cells extend processes and express glutamine synthetase, as do the 1^st^-generation neuronal precursors that comprise the niche. A small percentage of cells extracted from the hemolymph of crayfish also label with the niche cell marker glutamine synthetase. The findings that serotonin treatment in vivo increases the niche cell population, that serotonin is localized to the rim of the vascular cavity, and that serotonin receptors are found in hemocytes, are consistent with the behavior of these cells in niche co-cultures, where flooding the culture dish with serotonin or the 5-HT_2ß _receptor antagonist MMS interferes with their affinity for the niche. For all of these reasons, we propose that the hematopoietic system is a potential source of primary neuronal precursor cells in crayfish. We do not know, however, whether cells recruited into the niche go through a gradual transformation to become resident bipolar niche cells that will at some later time become primary neuronal precursors (an "assembly line" or "inventory" model). This scenario would suggest that the niche cells represent progressive stages in the transformation and activation to 1^st^-generation neuronal precursors. Alternatively, newly recruited cells may be fast-tracked through the niche, escorted by the niche cells, as they progress through the cell cycle and divide (a "conveyor belt" or "just-in-time" model). Experiments designed to differentiate between these alternatives, as well as in vivo studies to track labeled hemocytes over periods of several days or weeks, are currently underway. By either scenario, however, the studies of Ayub et al. [38] show that the numbers of niche cells actively engaged in the cell cycle can be adjusted, presumably in response to local signals that are, in turn, influenced by the animal's living conditions. These findings confirm that the 1^st^-generation neuronal precursors are not single (or a few) specially-endowed cells, but rather a dynamic population whose proliferative potential is regulated.

The idea that hematopoietic stem cells may play a central role in adult neurogenesis is not a new proposal. Mammalian bone marrow cells have a proclivity to migrate to the brain when infused into a host animal [42-44]. In studies where bone marrow cells were grafted into the lateral ventricle they migrated throughout the brain, including areas undergoing active postnatal neurogenesis. The descendants of these cells express a variety of glial and neuronal markers, including GFAP and F4/80 (a microglial marker) [42], NeuN and NSE [45], NeuN and GFAP [46], and MAP-2, NeuN and GFAP [47], and in some studies these cells developed the characteristics of astrocytes [43]. In vitro, bone marrow cells have been induced by various means to form neurons [48-52], and in one study the bone marrow-derived neurons responded to depolarizing stimuli, showing a rapid and reversible calcium increase in response to acetylcholine [52], a response characteristic of neurons. While the identities of specific types of bone marrow cells that are the source of neurons and glia in these studies is not always clear, other experiments specifically used hematopoietic cells, and found that in the brain these could acquire neural features and express neural genes [53,54]. However, additional studies suggested that cell fusion may account for the acquisition of such broad properties by stem cells [55-58]. Nevertheless, the idea persists in the literature that cells derived from bone marrow can transdifferentiate into neuronal and glial precursors in response to signals in the brain.

## Conclusions

Our conclusion from these studies is that the 1^st ^generation neuronal precursor cells comprising a neurogenic niche in the adult crayfish brain are not self-renewing. The corollary of this finding is that these neuronal precursor cells must be replenished from a source extrinsic to the niche. Our in vitro data point to the hematopoietic system as one possible source of neuronal precursor cells, a finding that, if confirmed in further in vivo studies, has profound mechanistic, developmental and evolutionary implications.

## Abbreviations

5-HT: 5-hydroxytryptamine (serotonin); BrdU: 5-bromo-2-deoxyuridine; CTG CellTracker™ Green; EdU: 5-ethynyl-2'-deoxyuridine; GS: glutamine synthetase; LPZ: lateral proliferation zone; MMS: methiothepin mesylate salt; MPZ: medial proliferation zone; PCPA: *p*-chlorophenylalanine;

## Authors' contributions

JLB conceived the study, designed and executed most of the experiments, and participated in data interpretation and in manuscript preparation. YZ conducted the studies for Figure 2A and the protein analysis for Figure 6C-D, participated in experimental design, in data interpretation and manuscript preparation. CRK conducted the experiment described in Figure 1C, and provided technical support for other experiments and manuscript preparation. DCS participated in the design of the study and executed the experiments for dye injection of the vascular system. BSB carried out experiments for Figure 6A-B and was involved in the overall design of the study, data interpretation and manuscript preparation. All authors have read and approved the final manuscript.
